# Quality, Safety and Biological Studies on *Campylanthus glaber* Aerial Parts

**DOI:** 10.3390/ph16101373

**Published:** 2023-09-28

**Authors:** Katelene Lima, Maryam Malmir, Sérgio P. Camões, Kamrul Hasan, Samuel Gomes, Isabel Moreira da Silva, Maria Eduardo Figueira, Joana P. Miranda, Rita Serrano, Maria Paula Duarte, Olga Silva

**Affiliations:** 1 Research Institute for Medicines (iMed.ULisboa), Faculty of Pharmacy, Universidade de Lisboa, 1649-003 Lisbon, Portugal; k.lima@edu.ulisboa.pt (K.L.); m.malmir@edu.ulisboa.pt (M.M.); sergiocamoes@edu.ulisboa.pt (S.P.C.); mdkamrul@edu.ulisboa.pt (K.H.); icsilva@ff.ulisboa.pt (I.M.d.S.); efigueira@ff.ulisboa.pt (M.E.F.); jmiranda@ff.ulisboa.pt (J.P.M.); rserrano@edu.ulisboa.pt (R.S.); 2 Instituto Nacional de Investigação e Desenvolvimento Agrário (INIDA), São Jorge dos Orgãos, Santiago CP 84, Cabo Verde; samuel.gomes@inida.gov.cv; 3 The Mechanical Engineering and Resource Sustainability Center (MEtRICs), Nova School of Science and Technology, Universidade Nova de Lisboa, 2829-516 Caparica, Portugal; mpcd@fct.unl.pt

**Keywords:** antioxidant, antihyperglycemic, *Campylanthus glaber*, herbal drug, herbal medicine, quality control, safety, verbascoside

## Abstract

In Cabo Verde, several endemic species are used in traditional medicine. However, no scientific studies have been conducted on the quality, efficacy, and safety of most of these plants. This study focused on establishing the botanical and chemical identification parameters required for a quality monograph of *Campylanthus glaber* Benth. aerial parts, a medicinal plant of Cabo Verde traditionally used to treat fever and muscular pain. In addition, in vitro antioxidant and antihyperglycemic activity, cytotoxicity, and genotoxicity were assessed for this medicinal plant. Optical microscopy, LC/UV-DAD-ESI/MS, and colorimetric assays were used for botanical, chemical, and biological studies, respectively. Cytotoxicity was assessed by the MTT assay with HepG2 cells, and genotoxicity by the Ames test. Microscopically, the xeromorphic leaf of *C. glaber* presents a thick cuticle (13.6–25.5 µm), thick-walled epidermal cells, anomocytic-type stomata, glandular trichomes (stalk length = 49.4–120.8 µm), and idioblasts containing calcium oxalate microcrystals. The chemical screening of aqueous and hydroethanolic extracts of this medicinal plant revealed the presence of organic acids, iridoids, phenylethanoids, and flavonoids as the main classes of marker compounds, with malic acid, citric acid, and verbascoside being the main marker compounds identified. Both extracts showed similar LC/UV-DAD/ESI-MS qualitative profiles and DPPH radical scavenger activity (IC_50_ = 130.9 ± 1.4; 134.3 ± 3.1 µg/mL). The hydroethanolic extract inhibited both *α*-amylase and *α*-glucosidase enzymes in a dose-dependent manner. Both extracts showed no cytotoxicity (up to 1000 µg/mL) by the MTT assay and no genotoxic potential with or without metabolic activation up to 5 mg /plate. The results obtained are an important contribution to the monographic quality assessment of *C. glaber* aerial parts and suggest that this medicinal plant may be safe and potentially used as an herbal drug raw material for pharmaceutical purposes.

## 1. Introduction

*Campylanthus glaber* Benth. is a species endemic to the Cabo Verde archipelago that belongs to *Plantaginaceae* Juss., a highly heteromorphic family containing 107 genera and around 2000 species [1]. The genus *Campylanthus* Roth was taxonomically controversial for several years and was for a long time included in the family *Scrophulariaceae* Juss. and in the tribes *Digitaleae* and *Veroniceae* [2]. However, after molecular phylogenetic analysis, the genus was identified as related to *Globularia* Tourn ex L. and included in the *Plantaginaceae* family [3].

The genus *Campylanthus* includes 18 species with a wide distribution; 16 species occur from northeast Africa to southern Arabia and Pakistan, and the two other species (*Campylanthus salsaloides* (L.f.) Roth and *Campylanthus glaber*) are restricted to Macaronesia (Canary Islands and Cabo Verde Islands, respectively) [3,4]. In Cabo Verde, the species is distributed across the islands of Santo Antão, São Vicente, São Nicolau, Brava, Fogo, and Santiago, where it grows on cliffs and rocky and sandy slopes [4,5].

**Figure 1:**
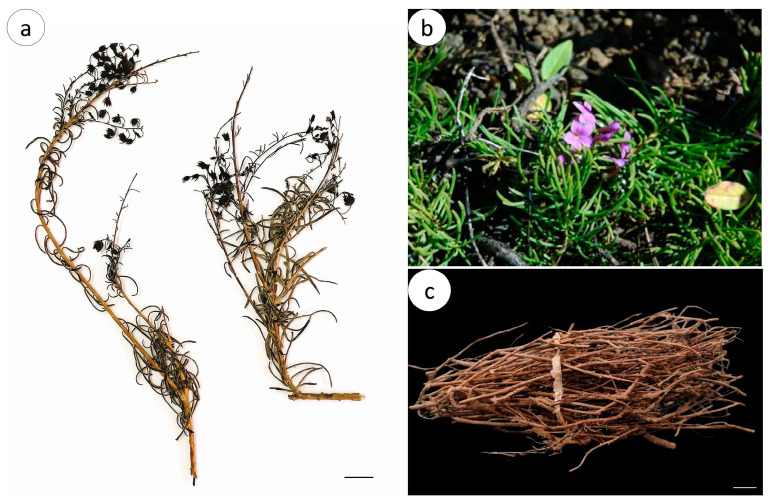
Macroscopic characteristics of *Campylanthus glaber* aerial parts; (**a**) *C. glaber* dried aerial parts; (**b**) *C. glaber* flowering branch; (**c**) Bunch of dried aerial parts of *C. glaber* (alecrim-bravo) sold in Santiago Island market (Praia market); scale bars: 1 cm. Photographies by Katelene Lima (**a**,**b**) and Maria Cristina Duarte (**c**).

*Campylanthus glaber* (Figure 1) is a shrub that is moderately branched, decumbent, or ascending to erect and grows up to 1 m high. The branches are puberulent to hirsute and leafy, with persistent leaf bases in older parts. The leaves are linear and narrowly oblong to spathulate, 2–5 cm long and 0.1–0.9 cm wide, more or less succulent, glabrous to hirsute, with apex acute to obtuse, and entire margin [5].

According to Plants of the World Online, *Campylanthus glaber* is the currently accepted name for this species. *Campylanthus glaber* is a highly variable species, and for this reason, several morphologically different populations of this species were frequently described as distinct species, subspecies, and varieties. However, they are currently recognized as synonyms. Nowadays, *Campylanthus benthamii* Webb, *Campylanthus benthamii* var. *glaber* (Benth.) Webb, *Campylanthus benthamii* var. *hirsutus* Webb, *Campylanthus glaber var. puberulus Cout.*, *Campylanthus glaber var. pumilus Pett.*, *Campylanthus glaber* subsp. *spathulatus* (A.Chev.) Brochmann, N.Kilian, Lobin & Rustan., and *Campylanthus spathulatus* A.Chev. are considered homotypic and heterotypic synonyms of *C. glaber* [1].

*C. glaber*’s chemical composition is sparsely studied. Ronsted and Jensen (2002) [2] analyzed fresh aerial parts of *C. glaber* ethanolic extracts and identified, by NMR and comparison with literature data, two main classes of compounds: iridoid glucosides (aucubin, erigeroside, gardoside, monomellithoside, melittoside, geniposidic acid, and 8-epilogonic acid) and caffeoyl phenylethanoid glycosides (salidroside and lavandulifolioside). In *Campylanthus salsaloides*, fresh aerial parts ethanolic extract similar compounds (erigeroside, aucubin, gardoside, monomellithoside, geniposidic acid, lavandulifolioside), were identified in addition to mussaenosidic acid, mannitol, and sorbitol, [2].

Aerial parts of *C. glaber*, commonly known as “alecrim-brabo”, are used in Cabo Verde to treat muscular pain [6], headache and menstrual problems [7], fever, flu, and congestion [8], and the whole plant is also used as food, animal feed (pasture), and wood fuel [8].

The estimated percentage of the African population’s reliance on traditional forms of medicine to satisfy their healthcare needs is still extremely high [9]. In Cabo Verde, traditional medicine plays an important role in the health system of the country since rural communities still have limited access to conventional medicine [10,11]. 

While in Africa traditional medicines and herbal medicines continue to be widely used, the world is gradually turning back to herbal medicines to treat multiple diseases and ailments with more affordable costs and a greater degree of safety (less side effects) compared to conventional drugs [12].

Plants are an extensive source of natural products [13] with broad pharmacological potential [14]. Over millions of years, plant secondary metabolites have evolved and acquired a unique chemical diversity, which subsequently led to a variety of biological activities and drug-like properties [15]. Therefore, even after all the advances in drug development and the globalization of modern medicine, plants and herbal medicines continue to be one of the most important raw materials for medicines and continue to play a central role in healthcare systems around the world [16].

*C. glaber* is listed as an endangered (EN) species on the International Union for the Conservation of Nature’s (IUCN) Red List of Threatened Species and is likely to be critically endangered. The species is included in the protected areas [17,18]. However, more strategies for its conservation are of crucial importance. The cultivation and valorization of medicinal plants as raw materials for pharmaceuticals [19] are some strategies that could be explored. Hence, with the present study, we aimed to obtain quality, efficacy, and safety data on *C. glaber* aerial parts, useful to allow its appropriate use as an herbal medicine according to official monographic quality parameters for herbal products.

## 2. Results

### 2.1. Botanical Quality Monographic Studies

#### 2.1.1. Macroscopic Characterization

*C. glaber* dried aerial parts (Figure 1a–c) are characterized by the presence of dried and fragmented leaves and stems with variable lengths.

**Figure 2:**
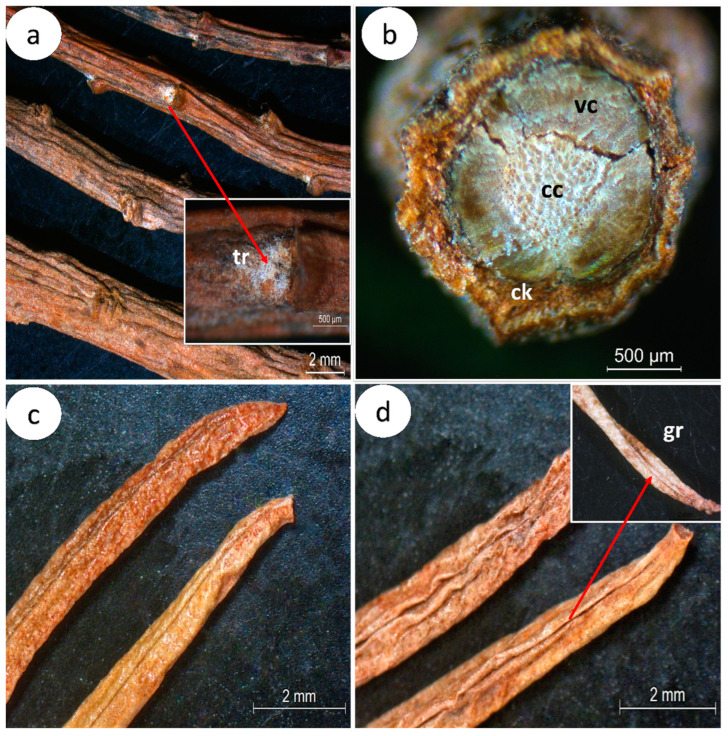
Macroscopic characteristics of *Campylanthus glaber*, aerial parts: (**a**) general aspect of the stem branches with the presence of trichomes (arrow) in the younger branch nodes; (**b**) cross-section section of the stem branch with details of the cork region and vascular cylinder; (**c**) leaf abaxial view; (**d**) leaf adaxial view with detail of groove (arrow) formed by revolute margin covered by trichomes. Abbreviations: cc: central cylinder; ck: cork; vc: vascular cylinder; tr: trichomes.

Macroscopically, the *C. glaber* stem is dark brown, cylindrical, decumbent to erect, moderately branched, with longitudinal striation, alternate nodes, and rarely hirsute branch nodes (Figure 2a). The *C. glaber* stem presents three distinct regions: the periderm, cork, and vascular cylinder (Figure 2b). The dried leaf is dark brown, linear to narrowly obovate, 1–5 cm long and 3–10 mm wide, with an acute apex, tubular base, revolute margin forming a longitudinal groove covered by trichomes (Figure 2c,d), and an indistinct venation pattern.

#### 2.1.2. Microscopic Characterization

A microscopy examination of the *C. glaber* stem, leaf, and aerial parts powdered drug was carried out by light optical microscopy. The quantitative analysis of the different anatomical features identified in both the leaf and stem is presented in Table 1.

##### Stem

**Figure 3:**
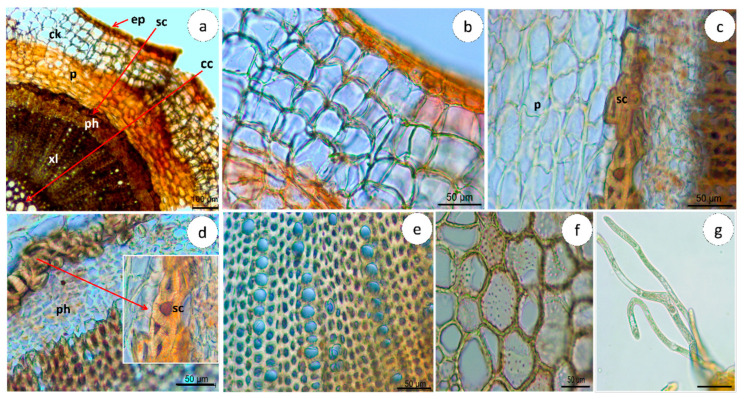
Microscopic characteristics of the *Campylanthus glaber* stem cross-section. (**a**) General aspect of the epidermis (arrow (ep)), cork, parenchyma, sclerenchyma (arrow (sc)), phloem, xylem, and central cylinder (arow(cc)) (from outside to inside). Details of (**b**) cork with four layers of quadrangular-shape cells; (**c**) parenchyma and groups of sclereids; (**d**) groups of sclereids forming a ring around the phloem; (**e**) xylem vessels organized in 2–8 cells; (**f**) central cylinder large thick-walled cells; (**g**) trichomes located in the epidermis. Abbreviations: cc: central cylinder; ck: cork; ep: epidermis; p: parenchyma; ph: phloem; sc: sclereids; xl: xylem. Scale bars: (**a**) = 100 µm; (**b**–**g**) = 50 µm.

In cross-sections, the stem shows a typically cylindrical format (Figure 2b and Figure 3a), organized into the periderm, cork, and vascular cylinder (Figure 3a) from outside to inside. In the periderm, the phellem region of about 4–7 rows of quadrangular-shape cork cells (Figure 3b, Table 1), followed by a parenchyma region (Figure 3a), is below a one-layer epidermis covered with a striate cuticle. The cortex region is characterized by the presence of a circular layer of grouped sclereids (sclerenchyma) with thick walls (3.28–10.17 µm) (Figure 3a–c, Table 1) surrounding the vascular cylinder, mainly occupied by a broad xylem with 2–8 rows of vessels (82.1–324.1 µm^2^) (Figure 3a–e, Table 1), a narrow phloem (Figure 3a–d), and a central cylinder with large (770.9–4340.5 µm^2^) thick-walled cells (Figure 3f, Table 1). Few trichomes were observed; however, they are rarely present (Figure 3g).

##### Leaf

**Figure 4:**
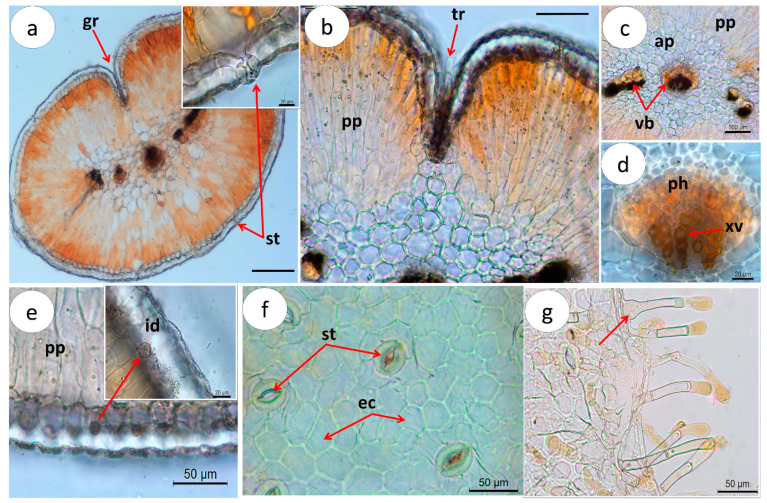
Microscopic characteristics of *Campylanthus glaber* leaf. (**a**) Cross-section of the midrib showing the cylindrical mesophyll with detail of stomata (arrow (st)) and groove (arrow (gr)); (**b**) detail of trichome (arrow) located in the groove formed by the curved margins and palisade parenchyma; (**c**) vascular bundles (arrows) embedded in the aquifer parenchyma; (**d**) xylem vessels (arrow) and phloem; (**e**) idioblasts (arrow) with calcium oxalate microcrystals; (**f**) epidermal cells (arrows (ep)) and anomocytic-type stomata (arrows (st)); (**g**) trichomes located on the leaf margins with detail of trichomes with swollen base (arrow). Abbreviations: ap: aquifer parenchyma; ec: epidermal cells; gr: groove; id: idioblast; pp: palisade parenchyma; ph: phloem; st: stomata; vb: vascular bundle; xv: xylem vessel. Scale bars: (**a**) = 200 µm; (**b**,**c**) = 100 µm; (**d**) = 20 µm; (**e**–**g**) = 50 µm.

*C. glaber* leaf shows a circular shape in cross-section, with revolute margins forming a longitudinal groove over the upper surface. The leaf is characterized as monofacial since the epidermis consists of one single layer of epidermis covered by a thick striated cuticle (13.7–25.6 µm) (Table 1), continuous from the upper to the lower surface (Figure 4a). Stomata inserted around the cylindrical mesophyll and glandular trichomes (Figure 4a,b), restricted to the groove formed by the leaf revolute margins, were observed.

*C. glaber* cylindrical mesophyll is differentiated into a strongly developed layer of palisade parenchyma organized in a tightly packed thick multilayer (154–323µm) of 3–5 columnar cells arranged perpendicularly pointing towards the center (Figure 4a,b, Table 1) and water-storage parenchyma (aquifer parenchyma), consisting of 3–4 layers of large, thick-walled cells located in the central part of the mesophyll (Figure 4c).

The leaf vascularization pattern, observed after a cross-section, revealed the presence of a large central bundle and a variable number of small lateral vascular bundles embedded in the aquifer parenchyma (Figure 4c). The xylem, organized in rows of 2–4 vessels (Figure 4d), is oriented towards the upper surface, with angular collenchyma occurring around the largest bundle (Figure 4d). Calcium oxalate microcrystals (sands) grouped in idioblasts dispersed in the epidermis were also observed (Figure 4e).

Paradermal sections revealed rectangular to polygonal epidermis cells with variable length (22.1–55.4 µm), anomocytic-type stomata (subsidiary cells like epidermal cells) (Figure 4f), and multicellular, uniseriate capitate glandular trichomes, constituted by a bicellular to multicellular pedicel with one or two cells and a unicellular secretory head. The trichomes distinctively swollen bases were also observed (Figure 4g).

##### Powdered Herbal Drug

**Figure 5:**
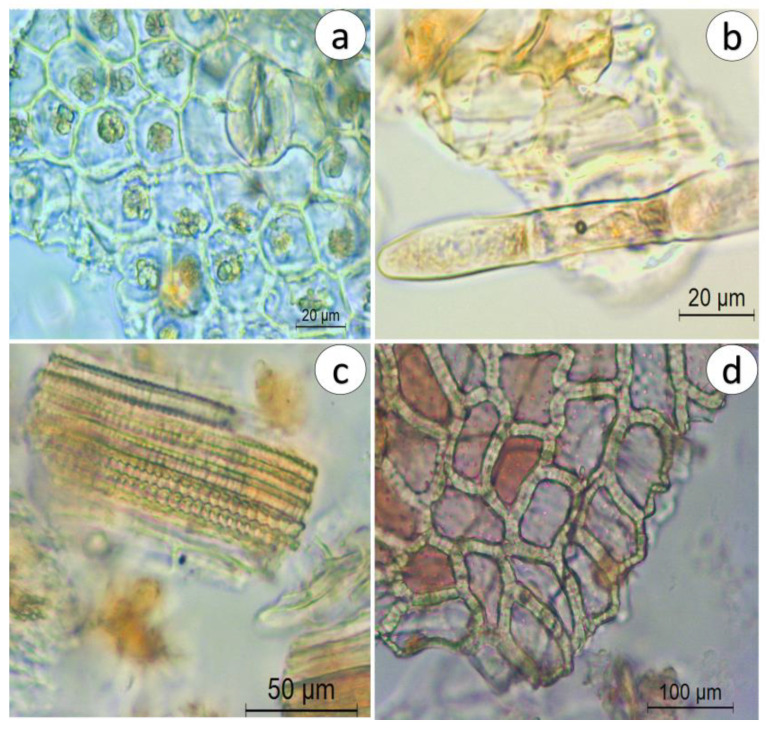
Microscopic characteristics of *C. glaber* aerial part powder. (**a**) Fragmented leaf epidermal cells with stomata and idioblasts with microcrystals; (**b**) protective trichome; (**c**) fragment of pitted vessels; (**d**) fragment of the stem central cylinder.

The powder drug is dark brown and is characterized by the presence of typical structures of both leaf and stem. The most common structures observed are isolated anomocytic stomata, idioblasts with microcrystals, sclereids, fragmented leaf epidermal cells with stomata and clustered crystals, glandular (Figure 5a) and protective trichomes (Figure 5b), pitted vessels (Figure 5c), and the stem central cylinder (Figure 5d).

### 2.2. Chemical Characterization

#### 2.2.1. Drug–Extract Ratio

The drug–water extract (CgAE) ratio was 4.39:1 and the drug-70% ethanol extract (CgEE) ratio was 2.89:1. The yield for CgAE was found to be 22.75% and 34.48% for CgEE.

#### 2.2.2. LC-UV/DAD Fingerprinting

**Figure 6:**
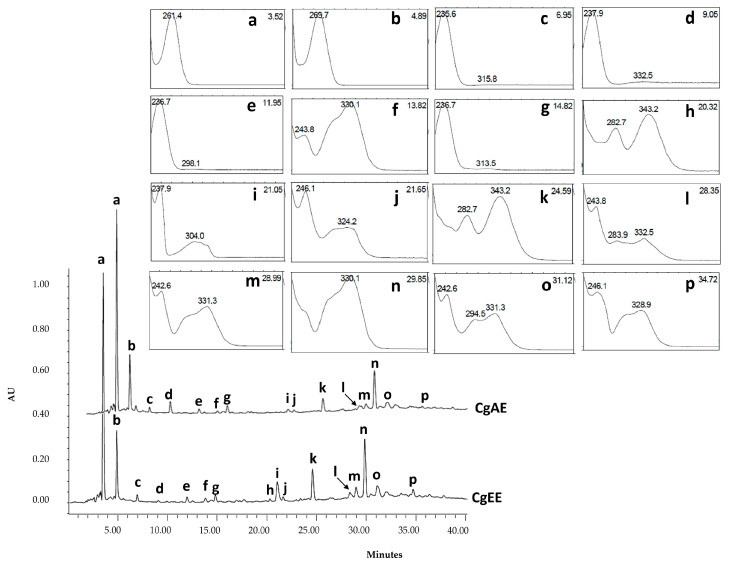
LC/UV-DAD profile of CgAE and CgE with UV spectra and retention times of the main marker classes of compounds identified: organic acids (peaks (**a**,**b**)), iridoid glucosides (peaks (**c**–**e**,**g**)), caffeoyl phenylethanoids glycosides ((**f**,**j**,**l**–**p**)) flavonoids (peaks (**h**,**k**)), and an unknown compound (**i**). Abbreviations: CgAE: *Campylanthus glaber* aqueous extract; CgEE: *Campylanthus glaber* ethanolic extract.

The liquid chromatography coupled with ultraviolet photodiode array (LC-UV/DAD) profiles obtained for CgAE and CgEE are presented in Figure 6. Observation of the chromatographic characteristics (UV spectra and retention times) permitted the tentative identification of the 16 major peaks as organic acids (Figure 6a,b), iridoid glucosides (Figure 6c–e,g), caffeoyl phenylethanoids glycosides (Figure 6**f**,j–p), flavonoids (Figure 6h,k) and an unknown compound (Figure 6i).

#### 2.2.3. LC-UV-ESI/MS-MS Chemical Profile

The identity of the main marker compounds detected in *C. glaber* extracts was tentatively made through the comparison of retention times *(t*_r_), maximum wavelengths (*λ*_max_), pseudo-molecular ion ([M-H], and corresponding fragment ions (MSn) with those of reference standards and of published literature data (Table 2).

Peak **a** with *λ*_max_: 261 nm, precursor ion [M-H]^−^ at *m*/*z* 133, and characteristic fragment at *m*/*z* 115 corresponding to [M-H-H_2_O]^−^ was proposed as malic acid. Peak **b** with *λ*_max_: 269 nm, precursor ion [M-H]^−^ at *m*/*z* 191, and characteristic fragment at *m/z* 111 corresponding to [M-H-CO_2_-2H_2_O]^−^ was proposed as citric acid [20,21].

The losses of H_2_O (18 Da), CO_2_ (44 Da), and glucosyl moiety (Glc,162 Da) were detected frequently in iridoids. Peak **c** with *λ*_max_: 234 and peak **d** with λ_max_: 238 and precursor ions [M-H]^−^ at *m*/*z* 373 and characteristic fragment at *m*/*z* 149 corresponding to [M-Glc-CO_2_-H_2_O-H]^−^ were proposed as gardoside and geniposidic acid, respectively [22,23]. The geniposidic acid identity was also confirmed by co-chromatography with a reference standard [22,23].

Peak **e** with *λ*_max_: 235, precursor ion [M-H]^−^ at *m*/*z* 375 and characteristic fragment at *m*/*z* 169 and *m*/*z* 151 corresponding to [M-Glc-CO_2_-H]^−^ and [M-Glc-CO_2_-H_2_O-H]^−^, respectively, was proposed as 8-epiloganic acid [23,24].

In phenylethanoid glycosides, the frequent neutral losses are rhamnose (162 Da) and caffeoyl moiety (146 Da). In addition, fragments at *m*/*z* 179 and 161 are also detected frequently, as they are produced by caffeic acid and glucose. Peak **f** with a *λ*_max_: 244, 330; precursor ion [M-H]^−^ at 487; and characteristic fragment at *m/z* 341 corresponding to [M-caff-H]^−^ was proposed as a cistanoside isomer.

Peak **j** with *λ*_max_: 246, 283sh, 324 nm; precursor ion [M-H]^−^ at *m*/*z* 639; and characteristic fragment at *m*/*z* 621, corresponding to [M-H-H_2_O]^−^ and the fragments *m*/z 161 and 179, belonging to the caffeoyl moiety, were proposed as a *β*-hydroxyverbascoside isomer.

Peak **m** with *λ*_max_: 330, precursor ion [M-H]^−^ at *m*/*z* 755, and characteristic fragment at *m*/z 593, corresponding to [M-H-rhamnose]^−^, was proposed as lavandulifolioside [25]. 

Peaks **n**, **o,** and **p** with a *λ*_max_: 330, 331, and 328 nm, respectively; precursor ion [M-H]^−^ at *m*/*z* 623; characteristic fragment at *m*/*z* 461, corresponding to [M-H-Glc]^−^; and the fragments *m*/z 161 and 135 belonging to the caffeoyl moiety were proposed as verbascoside, *cis*-verbascoside, and isoverbascoside, respectively [23,26].

Additionally, three other iridoid glucosides, with precursor ions [M-H]^−^ at *m*/*z* 361, 345, and 523 and characteristic fragments at *m*/*z* 407, 391, and 593, respectively, corresponding to [M+HCOOH]^−^ [23,27], were detected only by LC-UV-ESI/MS-MS. These compounds were proposed as melitoside, aucubin, and monomelitoside, respectively. Aucubin’s identity was also confirmed by co-chromatography with an authentic reference standard.

#### 2.2.4. Quantification of Secondary Metabolites

The results of the total polyphenolic content (TPC), total flavonoid content (TFC), and total iridoid content (TIC) of CgAE and CgEE are presented in Table 3. CgEE exhibited a higher content of TPC (148.1± 9.5 mg GAE/g) and TFC (75.8 ± 0.2 CE/g) when compared to CgAE (131.3 ± 3.9 mg GAE/g, 71.0 ± 0.6 CE/g, respectively). CgEE exhibited a slightly higher value of TIC compared to CgAE (6.1 ± 1.3 and 4.9 ± 0.6 mg Au/g, respectively), butHowever, with no statistical significance (*p* value: 0.1074).

Overall, CgEE exhibited a higher yield, drug–extract ratio (DER), and higher content of the main classes of secondary metabolites quantified.

### 2.3. In Vitro Biological Studies

#### 2.3.1. In Vitro Antioxidant Activity

The results of the evaluation of CgAE and CgEE antioxidant potential by the CUPRAC (cupric-reducing antioxidant capacity), FRAP (ferric-reducing antioxidant power), and DPPH (2,2-diphenyl-1-picrylhydrazyl) assays are presented in Table 4.

The obtained CUPRAC values ranged from 197.9 ± 2.6 to 203.8 ± 1.8 µg/g for both tested extracts, with CgEE presenting significantly (*p* value: 0.017) higher reducing ability than CgAE. For the FRAP assay, CgAE (109.8 ± 3.2 µg/g) displayed slightly higher reducing capacity than CgEE (104.0a ± 3.9 µg/g); however, there was no statistical significance (*p* value: 0.058).

Obtained results for the DPPH radical scavenge assay showed that CgE with an IC_50_ value of 134.3 ± 1.4 μg/mL and CgA with an IC_50_ value of 130.9 ± 3.1 μg/mL had similar antioxidant potential (*p* value: 0.092) but were lower when compared to ascorbic acid used as a reference standard (IC_50_ = 17.3 ± μg/mL). Both extracts showed DPPH radical-scavenging activity in a dose-dependent manner.

#### 2.3.2. In Vitro Enzymatic Inhibition of Diabetes Type 2-Related Enzymes

The inhibitory activity of *C. glaber* aerial part extracts against *α*-amylase and *α*-glucosidase, two important enzymes related to the management of postprandial serum glucose levels in diabetes type 2, was investigated, and the results are depicted in Table 5.

Both CgAE and CgEE exhibited low inhibition activity against *α*-amylase (IC_50_: 7.21 ± 0.23 and 5.77 ± 0.11 mg/mL, respectively) when compared to the positive control acarbose (IC_50_: 0.011 ± 0.001 mg/mL). Regarding the *α*-glucosidase enzyme, only CgEE was able to inhibit this enzyme in the range of the tested concentrations. The inhibition potential (IC_50_: 827.9 ± 11.2 μg/mL), however, was moderate when compared to the positive control, acarbose (IC_50_: 350.3 ± 15.4 μg/mL). Both extracts inhibited the *α*-amylase enzyme in a concentration-dependent manner, while only the CgEE was able to inhibit the activity of the *α*-glucosidase enzyme.

### 2.4. Safety Assessment

#### 2.4.1. In Vitro Genotoxic/Mutagenic Potential

The genotoxic potential of CgAE and CgEE was evaluated by the Ames test. The criteria to consider a compound/substance mutagenic in the Ames test are the observation of a reproducible and significant increase (2–3 fold) in the number of revertant colonies per plate in at least one of the tester strains and a dose–response relationship between the concentrations evaluated [28,29,30].

Based on these criteria, the obtained results (Table 6 and Table 7) showed that CgAE and CgEE do not exhibit mutagenic/genotoxic potential, since none of these extracts induced an increase in the number of revertant colonies when compared to the negative control for any of the tester strains (TA98, TA100, TA102, TA1535, and TA1537), neither in the absence (Table 6) nor in the presence of metabolic activation (Table 7) (assay performed only for CgEE given the chemical similarities outlined in the previous section). Negative control values were according to the historical control data. As expected, the positive controls were able to meet the criteria of increasing more than twofold the mutation frequency when compared with the negative controls. The cytotoxicity evaluated in terms of a decrease in bacterial lawn background, a significant reduction in the number of revertant colonies, and/or the presence of microcolonies was not observed for any of the extracts at any of the tested concentrations.

#### 2.4.2. In Vitro Assessment of Cytotoxicity

**Figure 7:**
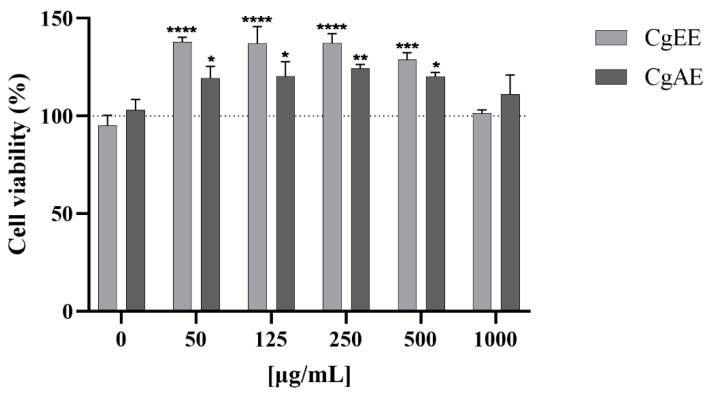
Viability of HepG2 after incubation (48 h) with CgAE and CgEE extracts assessed by MTT reduction assay. Data arpresented as percentage of solvent control (dashed line) and mean *±* standard deviation; *n* = 2–3. * *p* < 0.05; ** *p* < 0.01; *** *p* < 0.001; **** *p* < 0.0001.

The cytotoxicity of the *C. glaber* aerial part extracts was evaluated by the methylthiazolyldiphenyl-tetrazolium bromide (MTT) assay on HepG2 (a human liver cell line). Results expressed as cell viability percentage data are presented in Figure 7.

Neither CgAE nor CgEE cause cell death at any of the concentrations tested (50, 125, 250, 500, and 1000 µg/mL). On the other hand, an increase in the viability/proliferation of HepG2 was observed for both extracts at concentrations up to 1000 µg/mL when compared to the negative control (0 µg/mL). CgAE induced up to an approximately 24% increase in cell viability/proliferation, while CgEE induced an approximately 37% increase in viability/proliferation.

## 3. Discussion

There is an increasing demand for medicinal plants used as herbal drugs in traditional medicine worldwide. Therefore, the determination of botanical parameters of medicinal plants together with their chemical profiles is essential step in setting standards to determine the quality and authenticity of plant raw materials used as herbal drugs. This study focused on establishing macro- and micro-morphological parameters, phytochemical profiles, pre-clinical safety, and the pharmacological potential of *Campylanthus glaber* aerial parts.

The observed macroscopic and microscopic morphological characteristics of *C. glaber* leaf, stem, and aerial parts powder allowed identification of the most useful features for botanical monographic quality identification of this medicinal plant as an herbal drug. 

Macroscopic characteristics observed for *C. glaber* leaf morphology, such as linear to narrowly obovate shape, length and width (1–5 cm long and 3–10 mm wide), and acute apex, agree with the data reported by Brochman et al. (1994) and Hjertson (2003) [4,5] for this plant part. However, features like revolute margins were noticed for the first time in *C. glaber*, although already reported by Hjertson (2003) for *Campylanthus anisotrichus*, a species of the same botanical genus.

The present study is original and concerns the first microscopic characterization of *Campylanthus* species as herbal medicines, in particular *Campylanthus glaber* leaf and stem. In fact, apart from some studies on seed, pollen, and calix indumentum scanning electron microscopy [4,33], no studies regarding the micromorphology of these plant parts were found concerning other *Campylanthus* species. Therefore, the obtained results are considered fundamental for the identification of this medicinal plant (*C. glaber* aerial parts) as raw material for pharmaceutical purposes and for providing new elements that can facilitate the correct identification of other species of the same botanical genus. 

The most distinctive elements for the quality control are the leaf cylindrical shape in cross-section, the presence of strongly developed palisade parenchyma, the centrally located vascular bundles, the rectangular and polygonal thick-walled epidermal cells, the anomocytic type of stomata, glandular trichomes with a swollen base, and idioblasts with calcium oxalate crystals. Trichomes with swollen bases already described in the leaves of *C. antonii*, *C. junceus*, *C. pungens*, *C. ramosissimus,* and *C. yemenensis* [4] were observed for the first time in *C. glaber* leaf.

Concerning the stem microscopic characteristics, the cork layer with 3–7 rows of quadrangular-shape cells, thick-walled grouped sclereids surrounding the vascular cylinder with narrow phloem, broad xylem, and thick-walled central cylinder cells are the most distinctive features.

The macro- and microscopy examination of *C. glaber* leaf revealed xeromorphic morphological and anatomical adaptations. In fact, according to Hjertson 2003, most *Campylanthus* species show quite obvious xerophytic adaptations, and in *C. glaber* leaves, this is confirmed by the presence of small and narrow cylindrical leaves, succulence, thick cuticles, thick-walled epidermal cells, mesophyll mainly occupied by highly developed palisade parenchyma, and trichomes confined to a protected groove.

All of these traits can be related to the species adaptation to an habitat that constantly suffers from water stress, such as the Cabo Verde archipelago [34]. The thickening of the cuticle increases the resistance to water loss through the leaf surface [35], the cylindrical, small, narrow leaf reduces the surface area to volume ratio, minimizing excessive leaf heating and consequently reducing transpiration [36], and the succulence is due to the presence of water storage tissue. Another important xeromorphic adaptation is the trichomes, confined to a protected area formed by the revolute margins. According to Crang et al. (2018) [37], plants with trichomes located in crypts (grooves), an area of high trichome density, form a kind of water vapor pocket that significantly reduces water loss through transpiration. In this study, the aqueous extract prepared by infusion simulated the traditional herbal preparation used in Cabo Verde, and to provide a more stable preparation, a 70% hydroethanolic extract was obtained by maceration, a common method of preparation used to obtain total extracts from plant raw materials, as it allows obtaining extracts with a more representative composition of the secondary metabolites present in the plant material. In fact, the extraction yield depends on the extraction method and the solvent used for extraction. The use of aqueous mixtures containing ethanol, methanol, acetone, and ethyl acetate provides good results in the extraction of natural products from plant matrices; however, ethanol is recognized as a good solvent for polyphenol extraction and is safe for human consumption [11,38].

The analysis of our results showed that the hydroethanolic extracts had slightly higher extraction yields and DERs than the aqueous extract, proving that the mixture of water and ethanol (30:70 *v*/*v*, T = 22 °C) was more efficient in extracting the phenolic compounds than water (T = 95 °C). Nevertheless, since the majority of the main marker compounds (organic acids, iridoids, and phenylethanoid glycosides) are also soluble or partially soluble in water, the differences observed were not particularly pronounced, as observed in LC-UV/DAD fingerprinting and quantification of marker compounds (Figure 6, Table 3).

Phytochemical investigation of *C. glaber* aerial part extracts enabled the tentative identification of compounds belonging to organic acids, iridoid glycosides, phenylethanoid glycosides, and flavonoids. Based on their MS and MS/MS spectral data in negative ion mode together with relevant literature data and the use of authentic standards (co-chromatography), fourteen compounds, namely, malic acid (a), citric acid (b), gardoside (c), geniposidic acid (d), 8-epiloganic acid (e), cistanoside isomer (f), β-hydroxyverbascoside isomer (j), lavandulifolioside (m), verbascoside (n), cis-verbascoside (o), and isoverbascoside (p), in addition to the compounds monomelitoside, aucubin, and melitoside, were identified as major marker compounds of *C. glaber* extracts. 

Iridoid glycosides and phenylethanoid glycosides have been previously reported in the *Campylanthus* genus [2,39], where they present important chemotaxonomy values. The presence of aucubin, gardoside, lavandulifolioside, and melittoside shows the chemotaxonomic relationship of *Campylanthus* with *Plantago* L. [2,40]. Although chemotaxonomy studies *on Campylanthus* have been documented, detailed phytochemical studies of other *Campylanthus* species are scarce. According to Ronsted and Jensen (2002) [2], the chemical profiles of *C. glaber* and *C. salsaloides* differ only by the presence of 8-epiloganic acid and salidroside in *C. glaber* and mussaenosidic acid in *C. salsoloides*. Salidroside, as well as erigeroside, previously detected in both species, were not detected in the present study. On the other hand, this is the first report of the phenylethanoid glycosides *β*-hydroxyverbascoside isomer, verbascoside, cis-verbascoside, and isoverbascoside, as well as the organic acids citric acid and malic acid, in *C. glaber*. 

Regarding the quantification results of the main marker secondary metabolites, phenolic compounds, namely, phenylethanoid glycosides, are the main chemical classes of *C. glaber* aerial part extracts. Although iridoid glucosides are present in significantly smaller amounts in both extracts, they may still contribute to the biological potential of these extracts, as in studies related to the correlation of chemical composition and biological studies of other Plantaginaceae species, namely, the *Plantago* genus, both phenylethanoids and iridoids are referred to as the major classes of chemical compounds responsible for their pharmacological activities [41,42].

The severity of many chronic diseases, such as diabetes mellitus, chronic inflammation, cardiovascular disease, or age-related neurodegenerative disorders, is influenced by the abundance of reactive oxygen species (ROS) and other free radicals in the body. The ability of antioxidants to scavenge ROS and other free radicals [43] can delay the progression of many diseases by minimizing redox imbalances and counteracting the oxidative damage to tissues and biological macromolecules, such as lipids, proteins, and DNA [42,44].

*C. glaber* aerial part extracts presented in vitro antioxidant potential in three different ways: CUPRAC, FRAP, and DPPH radical scavenging ability. Although both extracts showed similar antioxidant activity in the FRAP and DPPH assays, CgEE displayed slightly higher antioxidant potential in the CUPRAC assay, which may be related to a higher amount of phenolic compounds displayed by CgEE compared to CgAE.

The antioxidant activity displayed by both extracts may be mainly due to the presence of phenylethanoids and flavonoids, since phenolic compounds, particularly flavonoids, can directly scavenge free radicals by donating electrons or hydrogen atoms. The presence of an OH group in their structure is the most significant determinant of electron-donating activity [43,45]. Concerning the iridoids, although they have been reported to have great anti-inflammatory potential, they appear to contribute less to the antioxidant activity of *C. glaber* extracts compared to the other phenolic compounds, as their chemical structure has a low number of OH groups, making them poor donors of hydrogen [24]. In this study, citric and malic acids were the main organic acids present in *C. glaber* extracts. Previous studies have shown that organic acids’ effective antioxidant potential is due to their chelation capacity, which can inactivate reduced cations [46]. It has been reported that organic acids are able to decrease reactive oxygen species and increase the bioavailability of phenolic compounds. Hence, it is possible to suppose a synergistic effect of malic and citric acid and the phenolic compounds present in *C. glaber*’s antioxidant potential.

The potential of *C. glaber* aerial part extracts to inhibit enzymes related to diabetes type 2 was also investigated. Diabetes mellitus is a metabolic disorder characterized by chronic hyperglycemia [47,48]. One of the most common approaches to controlling postprandial hyperglycemia is the inhibition of key enzymes such as α-amylase and α-glucosidase, responsible for the breakdown of carbohydrate and polysaccharides into monosaccharides [49]. The inhibition of the activity of these enzymes helps decrease the rate of carbohydrate digestion, decreasing the rate of absorption of monosaccharides, thereby lowering postprandial serum glucose levels. CgEE exhibited higher enzymatic inhibition potential than CgAE. The observed differences between these two extracts may be related to the small differences observed in their quantitative chemical profiles. Although both extracts presented similar qualitative chemical profiles, CgEE exhibited a higher amount of phenolic compounds (flavonoids and phenylethanoid glycosides).

The phenylethanoid glycoside verbascoside (peak n in Figure 6), identified as the third major marker compound of these extracts, has been linked to the management of diabetes in studies with other medicinal plants. In fact, verbascoside and its isomers have shown inhibition potential against α-amylase and α-glucosidase enzymes [47,50]. In addition, a study by Gali et al. (2020) [51] found a dose-dependent protective effect of verbascoside against oxidative stress in clonal and human pancreatic β-cells, where dysfunction and failure are linked to the development of diabetes, therefore revealing the antioxidant potential of verbascoside as an important mechanism of action in diabetes management. Iridoid glucosides identified in *C. glaber* aerial part extracts, such as aucubin [52,53] and geniposidic acid [54], have also been associated with the management of diabetes and diabetes complications.

Acarbose, as an inhibitor of both α-amylase and α-glucosidase, is widely used to treat diabetes mellitus, but its clinical use is associated with side effects such as abdominal discomfort, bloating, diarrhea, and nausea. In addition, the use of acarbose is still limited in some developing countries due to its high cost [55]. The search for naturally abundant and safe sources of inhibitors of α-amylase and α-glucosidase is therefore of great relevance as a complementary/alternative treatment for diabetes type 2 [56].

Although the inhibitory potential of *C. glaber* aerial part extracts on both α-amylase and α-glucosidase enzymes was lower than that of acarbose, the obtained results showed that this medicinal plant is a natural source of natural products capable of inhibiting the activity of both enzymes in a dose-dependent manner. 

Medicinal plants are often assumed to be safe, even though this is not always true [57,58], as several studies are reporting the mutagenic potential of medicinal plants that have been used for many years [29,59,60], raising safety and economic concerns [61].

The Ames test and the cell proliferation assay (MTT assay) are recently being widely used in in vitro toxicology and genetic toxicology to predict the toxicity and genotoxic potential of plant extracts as preclinical safety assays on herbal drug production and to support the safe use of plants [29,61]. The Ames test uses Salmonella enterica Serovar Typhimurium LT-2 strains with mutations in the genes involved in histidine biosynthesis [30,62]. The histidine auxotrophic strains (his-) are able to detect gene mutations, specifically frameshift mutations and base pair substitutions [29]. The MTT assay is based on the ability of viable cells to reduce the MTT reagent, a yellow water-soluble tetrazolium salt, into purple formazan crystals through the succinate dehydrogenase system. The amount of purple formazan crystals produced is directly proportional to the number of viable cells in culture [63].

Results from the Ames assay showed that, tested up to the highest dose (5 mg per plate), *C. glaber* extracts did not induce a significant increase in the number of histidine revertant colonies over the negative control values obtained for tester strains TA 98, TA 100, TA 102, TA 1535, and TA 1537, neither in the presence nor in the absence of metabolic activation (Aroclor 1254-induced rat liver S9). In addition, the MTT assay results showed that *C. glaber* extracts did not induce cytotoxicity up to the highest dose tested (1000 µg/mL) in HepG2 cells when compared to the negative control, suggesting that neither CgAE nor CgEE may have toxic or genotoxic potential. Based on the obtained results, the use of *C. glaber* aerial parts in traditional medicine may pose no toxic or genotoxic risks for human consumption; however, further in vivo toxicity studies are an important future step to complete the safety profile of this medicinal plant.

## 4. Materials and Methods

### 4.1. Chemicals, Reference Items, and Reagents

*α*-Amylase from porcine pancreas, *α*-glucosidase from *Sacharomyces cerevisiae*, *α*-MEM, 2-aminoanthracene, 2-nitrofluorene, 2,2-diphenyl-1-picrylhydrazyl (DPPH), 2,4,6-tris(2-pyridyl)-s-triazine (TPTZ), 3,5-dinitrosalicylic acid, 4-nitrophenyl-*α*-D-glucopyranoside (NPG), 9-aminoacridine, aluminum chloride, benzo(a)pyrene, D-biotin, D-(+)-glucose monohydrate, glucose-6-phosphate, (+)-catechin, dimethyl sulfoxide, neocuproine, nicotinamide adenine dinucleotide phosphate, methylthiazolyldiphenyl-tetrazolium bromide (MTT), soluble potato starch, and *tert*-butyl hydroperoxide were purchased from Sigma-Aldrich (St. Louis, MO, USA). Ammonium sodium phosphate dibasic tetrahydrate, di-potassium hydrogen phosphate anhydrous, and sodium chloride were purchased from Fluka (Seelze, Germany). Bacto™ agar was obtained from Becton Dickinson & Co (Sparks, MD, USA). Acetic acid, ascorbic acid, citric acid monohydrate, disodium hydrogen phosphate dihydrate, hydrochloric acid, sodium acetate trihydrate, and sodium dihydrogen phosphate monohydrate were purchased from Panreac (Barcelona, Spain). Acetonitrile, Folin–Ciocalteu reagent, iron(III) chloride hexahydrate, L-histidine monohydrochloride monohydrate, methanol sodium carbonate, sodium hydroxide, and sodium nitrite were purchased from Merck (Darmstadt, Germany). Geniposidic acid was acquired from BLD Pharmatech GmbH (Kaiserslauterm, Germany). 4-Dimethylaminobenzaldehyde was acquired from Thermo Fisher Scientific^TM^ (Waltham, MA, USA). Acarbose and gallic acid were purchased from AlfaAesar (Karlsruhe, Germany). Ethanol was purchased from Carlo Erba Reagents (Val-de-Reuil, France. Ammonium acetate, copper (II) chloride dihydrate, and iron sulfate heptahydrate were purchased from Riedel-de Haën (Seelze, Germany); magnesium sulfate heptahydrate was purchased from LabChem Inc (Zelienople, PA, USA); nutrient broth nº 2 was purchased from Oxoid (Basingstoke, UK); and sodium azide was purchased from J.T. Baker Chemical Company (Phillipsburg, NJ, USA). Aucubin was acquired from Extrasynthese (Genay, France). Formic acid (99–100%) was from VWR (Radnor, PA, USA). Fetal bovine serum was purchased from FBS, Gibco^®^-Thermo Fisher ScientificTM (Waltham, MA, USA). Non-essential amino acids (NEAA) and sodium pyruvate were purchased from PAN Biotech (Aidenbach, Germany). Aroclor 1254-induced rat liver S9 was purchased from Trinova Biochem (GmbH, Giessen, Germany). A Milli-Q water purification system (Millipore, Molsheim, France) was used to obtain ultra-pure water.

### 4.2. Plant Material Collection and Processing

*C. glaber* aerial parts were collected at the Natural Park of Serra Malagueta, Santiago Island, Cabo Verde Archipelago, between April 2016 and May 2018. Botanical identification was performed by Samuel Gomes, and a voucher specimen (022016KL) has been preserved at the Herbarium of INIDA, Santiago, Cabo Verde. The fresh aerial parts were dried in the shade for one week.

### 4.3. Preparation of Extracts

The dried aerial parts of *C. glaber* were extracted with hot water (water extract, CgAE) and 70% ethanol (hydroethanolic extract, CgEE). CgAE was prepared through the infusion method by adding 200 mL of boiling ultra-pure water (95 °C) to 3 g of dried plant material. After 5 min of infusion and cooling, the extract was filtered, freeze-dried (Heto, LyoLab 3000), and stored. To prepare the CgEE, the dried plant material was previously ground into a homogenous powder using a grinder. This extract (1:10—1 part of the plant powder to 10 parts of solvent) was prepared by the maceration method at room temperature under agitation for 72 h. After filtration, the extract was evaporated under reduced pressure and a temperature of 38 ± 1 °C, freeze-dried (Heto, LyoLab 3000), and stored.

### 4.4. Botanical Characterization

#### 4.4.1. Macroscopic Evaluation

Macroscopic analyses were executed according to the methods of the European Pharmacopoeia (10th edition, 2019) [64]. The plant material was analyzed by naked eye and with an Olympus SZ61 stereomicroscope (Leica Microsystems Ltd., Heerbrugg, Switzerland) coupled with a Leica MC170 HD digital camera controlled by Leica Application Suite (LAS) Version 4.8.0 software (Leica Microsystems Ltd., Heerbrugg, Switzerland).

#### 4.4.2. Microscopic Evaluation

Microscopic analyses of dried leaves, stems, and aerial part powders were analyzed according to the European Pharmacopoeia (10th edition, 2019) [64]. An Olympus SZ61 stereo microscope coupled with a Leica MC170 HD digital camera for image capture was used. Image analysis was executed using Leica Application Suite (LAS) Version 4.8.0 software. Leaf (midrib) and stem microscopic analysis, freehand cross-section, and paradermal sections were analyzed. The samples were hydrated in distilled water and clarified with chloral hydrate (60%). The stoma index was determined using the following equation:

$$SI=\frac{St\times 100}{St+Ep}$$

where SI is the stomata index, St is the number of stomata, and Ep is the number of epidermal cells.

The dried powdered aerial parts of *C. glaber* were analyzed by placing small amounts of powder on a slide and observed under the microscope. Samples were mounted in chloral hydrate (60%). Characteristic structures and cell contents were observed.

All macro- and microscopic characterization results were expressed as mean ± standard deviation.

### 4.5. Chromatographic Conditions

The LC-UV/DAD analysis was performed with a Liquid Chromatograph Waters Alliance 2690 Separations Module (Waters Corporation, Milford, MA, USA) coupled with a Diode Array Detector (Water 966 PDA; Waters Corporation, Milford, MA, USA). The chromatograms were recorded at a maximum absorbance of each band (MaxPlot) between 210 and 450 nm, and Waters Millennium^®^ 32 Chromatography Software (Waters Corporation, Milford, MA, USA) was used for data analysis. A Purospher STAR RP-18 endcapped column (4 × 250 mm, 5 μm) with pre-column (4 × 250 mm, 5 μm) from Merck (Darmstadt, Germany) was used, and as mobile phase, a linear gradient of water (0.1% formic acid) (solvent A) and acetonitrile) (solvent B) (*v*/*v*) was used: 0 min, 5% A, 95% B; 12 min, 89% A, 11% B; 40 min, 0% A, 100% B. The flow rate used was 1 mL/min, and the column temperature was 25 ± 5 °C.

For LC-MS/ESI, the analysis was undertaken with an HPLC (Waters Alliance 2695), coupled with a photodiode array detector and an autosampler (Waters PDA 2996), in tandem with a triple quadrupole mass spectrometer (Micromass^®^ Quatro MicroTM API, Waters^®^, Ireland) and an electrospray ionization source (ESI) functioning in negative mode. A LiChrospher 100 RP-18 (5 µm) 250 × 4 mm column with a respective pre-column (Merck, Germany) was used. Water + 0.1% formic acid (solvent A) and methanol (solvent B) (*v*/*v*) were used as the mobile phase with a linear gradient: 0 min, 95% A, 5% B; 20 min, 80% A, 20% B; 60 min, 50% A, 50% B; 90 min, 0% A, 100% B. Acquired data were analyzed using MassLynx™ V4.1 software (Waters^®^, Ireland). The injection volume was 20 µL, the flow rate used was 0.3 µL/min, and the column temperature was 25 ± 5 °C.

All samples were prepared in 50% methanol at concentrations of 10 mg/mL for LC/UV-DAD analysis and at concentrations of 30 mg/mL for LC-MS/ESI analysis. The standards were prepared at concentrations of 1 mg/mL.

The identification of compounds was made based on a comparison of UV–Vis, retention time, and mass spectral data with those of standards injected in co-chromatography and/or with literature data.

### 4.6. Quantification of Main Classes of Secondary Metabolites

#### 4.6.1. Total Phenolic Content

Total phenolic content was estimated using the Folin–Ciocalteu colorimetric method described by Loebler et al. (2020) [65]. The absorbance was spectrophotometrically recorded at 765 nm (SPEKOL 1500, Analytik, Jena, Germany). Total phenolics amount was calculated using a gallic acid calibration curve (concentration range: 100–700 μg/mL; linear regression equation: y = 0.0896x + 0.0281; R² = 0.9986), and the results were presented as mg of gallic acid equivalents per g of dry extract (mg GAE/g).

#### 4.6.2. Flavonoid Content

Flavonoid content was estimated according to the method described by [66]. The absorbance was spectrophotometrically recorded at 510 nm (SPEKOL 1500, Analytik, Jena, Germany). The amounts of flavonoids were calculated using a catechin calibration curve (concentration range: 18–290 μg/mL; linear regression equation: y = 0.0032x + 0.0029, R² = 0.9991), and the results are presented as mg of catechin equivalents per g of dry extract (mg CE/g).

#### 4.6.3. Iridoid Content

Iridoid content was determined according to the method described by Niziol-Lukaszewska et al. (2017) [67]. The absorbance was spectrophotometrically recorded at 540 nm (Hitachi, U–2000, Tokyo, Japan). The amounts of iridoids were calculated using an aucubin calibration curve (concentration range: 10–200 µg/mL; linear regression equation: y = 0.0014x + 0.0119, R^2^ = 0.9906) and expressed as mg of aucubin equivalents per g of dry extract (mg Au/g).

### 4.7. Antioxidant Capacity

#### 4.7.1. CUPRAC Assay

The assay was performed following the method described by [68] with slight modifications. In a test tube, 1 mL of the following solutions were mixed: copper (II) chloride dehydrate (10 mM), ammonium acetate (1 M), and neocuproine in ethanol (7.5 mM). Then, 300 μL of extracts in appropriate dilutions were added to the previous mixture and water up to the final volume of 4.1 mL. After 1 h at room temperature, absorbance was spectrophotometrically recorded at 450 nm (SPEKOL 1500, Analytik, Jena, Germany). A calibration curve of ascorbic acid (concentration range: 2–45 µg/mL; linear regression equation: y = 0.025x + 0.057, R^2^ = 0.9966) was used, and the results were expressed as µg of ascorbic acid equivalents per g of dry extract (µg AAE/g).

#### 4.7.2. FRAP Assay

The assay was performed according to the procedure described by [69] with minor modifications. In test tubes, 100 µL of extracts of appropriate dilutions were mixed with 3 mL of freshly prepared and pre-warmed (37 °C) FRAP reagent (0.25 M sodium acetate buffer, pH 3.6, 10 mM TPTZ solution in 40 mM HCl, and 20 mM FeCl_3_. 6H_2_O at a ratio of 10:1:1). After incubation (4 min, 37 °C), the absorbance of the mixture was spectrophotometrically recorded at 593 nm (SPEKOL 1500, Analytik, Jena, Germany). A calibration curve of ascorbic acid (concentration range: 25–125 µg/mL; linear regression equation: y = 0.2692x + 0.0871, R^2^ = 0.9941) was used, and the results were presented as µg of ascorbic acid equivalents per g of dry extract (µg AAE/g).

#### 4.7.3. DPPH Radical Scavenging Assay

The ability of the samples to scavenge the DPPH radical was evaluated according to the method described by [70]. In test tubes, 500 μL of extract dilutions were mixed with 3 mL of freshly prepared DPPH solution (24 mg/L in ethanol). After 30 min of incubation in the dark at room temperature, the absorbance was measured at 517 nm (SPEKOL 1500, Analytik, Jena, Germany). Ascorbic acid was used as a positive reference. The scavenging activity was measured as the decrease in absorbance of the samples versus the DPPH standard solution. Results were expressed as inhibitory concentrations (IC_50_ values), representing the sample concentration required to scavenge 50% of the DPPH free radicals. The percentage of DPPH radical scavenging activity was calculated according to the formula:

$$Inhibition\left(\%\right)=\left[\frac{AbsControl-AbsSample}{AbsControl}\right]\times 100$$

### 4.8. Enzymatic Inhibition

#### 4.8.1. α-Amylase Inhibition Assay

The inhibition of the *α*-amylase enzyme was determined according to the method described by [71] with some modifications. Briefly, reaction mixtures containing 100 µL of Type VI-B porcine pancreatic *α*-amylase (0.5 mg/mL in 100 mM sodium phosphate buffer, containing 6.7 mM sodium chloride, pH 6.7) and 100 µL of extracts of appropriate dilutions were preincubated in test tubes at 37 °C for 10 min. Then, 100 µL of 1% starch (*w*/*v*, previously suspended in 100 mM sodium phosphate buffer, containing 6.7 mM sodium chloride, pH 6.7, and boiled for 10 min) was added. After 10 min at 37 °C, 200 µL of DNS reagent (consisting of 20 mL of 96 mM DNS, 8 mL of 5.315 M sodium potassium tartrate tetrahydrate in 2 M NaOH, and 12 mL of distilled water) was added. The reaction mixtures were heated at 100 °C for 15 min to stop the reaction, then cooled to room temperature and diluted with 2 mL of distilled water. The intensity of the redish color was spectrophotometrically measured at 520 nm (SPEKOL 1500, Analytik, Jena, Germany). The reaction mixture without the extracts was used as a negative control, and the reaction mixtures without *α*-amylase were used as samples’ blanks. Increasing concentrations of acarbose (10–125 µg/mL) were used as positive controls. The results were presented as the concentration in the reaction mixture that reduced the enzyme activity by 50% (IC_50_). The enzyme inhibitory rate was calculated according to the following formula:

$$Inhibition\left(\%\right)=\frac{\left(AbsSample-AbsSampleblank\right)\times 100}{AbsNegativecontrol}$$

#### 4.8.2. α-Glucosidase Inhibition Assay

The *α*-glucosidase enzyme inhibition was determined according to the colorimetric method described by [72], with some modifications. Reaction mixtures containing 5 µL of *α*-glucosidase (6.25 U/mL in phosphate buffer (pH 6.9, 0.1 M)), 125 µL of phosphate buffer (pH 6.9, 0.1 M), and 20 µL of the plant extracts at different concentrations were prepared in a 96-well microplate (Greiner Bio-One, Rainbach im Mühlkreis, Austria) and incubated for 15 min at 37 °C. The reaction was started by adding 20 µL of substrate solution (p-nitrophenyl-*α*-*D*-glucopyranoside, 2.75 mM in phosphate buffer (pH 6.9, 0.1 M)), and the plates were incubated for an additional 15 min at 37 °C. The reaction was stopped by the addition of 80 µL of 0.2 M Na_2_CO_3_. The absorbance of the wells was measured in a microplate reader (FLUOstar^®^ Omega Plate Reader, BMG 264 Labtech, Ortenberg, Germany) at 405 nm. The reaction mixture without the extracts was used as a negative control, and reaction mixtures without *α*-glucosidase were used as samples’ blanks. Increasing concentrations of acarbose (1–12 mg/mL) were used as positive controls. The results were expressed as the concentration in the reaction mixture that reduced the enzyme activity by 50% (IC_50_). The enzyme inhibitory rate was calculated as follows:

$$Inhibition\left(\%\right)=\frac{\left(AbsSample-AbsSampleblank\right)\times 100}{AbsNegativecontrol}$$

### 4.9. Safety Assessment

#### 4.9.1. In Vitro Assessment of Genotoxicity/Mutagenicity

The genotoxicity/mutagenicity potential of *C. glaber* extracts was evaluated by the Ames test, through the direct plate incorporation method described by Maron and Ames (1983) [30] and Mortelmans and Zeiger (2000) [62] and according to OECD No. 471 and ICH S2 (R1) guidelines [73,74]. The assay was performed with and without metabolic activation using the *Salmonella enterica* serovar Typhimurium (His^–^) tester strains TA98, TA100, TA102, and TA1535, provided by the Genetic Department of the Nova Medical School of the Universidade NOVA de Lisboa (Portugal) (received from Professor B.N. Ames (Berkeley, CA, USA)) and TA1537 (ATCC, NUMBER: 29630™ (LOT: 7405375)). Briefly, 200 μL of appropriate dilutions of the extracts in 30% DMSO (0–5 mg/plate), 100 μL of each tester strain (inoculated in nutrient broth medium and incubated in an orbital incubator for 12–16 h, 37 °C, 210 rpm in the dark, and kept at 4 °C until use) and 500 μL of phosphate buffer (0.1 M, pH 7.4) (assay without metabolic activation), or S9 mix (10%, *v/v* rat liver S9, 0.4 M MgCl_2_, 1.65 M KCl, 1 M glucose-6-phosphate, 0.1 M NADP in 0.1 M sodium phosphate buffer (pH 7.4) prepared fresh and kept on ice during the experiment) (assay with metabolic activation) were mixed with 2 mL of top agar (45 °C) supplemented with biotin (0.05 mM) and histidine (0.05 mM) and plated into minimal glucose agar medium (1.5% Bacto-Difco agar and 2% glucose in Vogel-Bonner medium). After 48 h of incubation at 37 °C, manual counting of revertant colonies (His+) was performed. 

Three independent experiments were performed for each assay, and the results were presented as the mean number of revertant colonies (His+) and the standard deviation (mean ± SD). The positive controls in the assay without metabolic activation were sodium azide (1.5 µg/plate for TA100 and TA1535), 2-nitrofluorene (5 µg/plate for TA98), 9-aminoacridine (100 µg/plate for TA1537), and tert-butyl hydroperoxide (50 µg/plate for TA102). The positive controls in the assay with metabolic activation were 2-aminoathracene (2 µg/plate for TA98 and 10 µg/plate for TA102, TA1535, and TA1537) and benzo(a)pyrene (5 µg/plate for TA100) in the assay with metabolic activation.

#### 4.9.2. In Vitro Assessment of Cytotoxicity

The cytotoxicity of the *C. glaber* extracts was evaluated by the MTT reduction assay [75] on the human liver cell line HepG2 (ATCC Cat. No. HB-8065, Middlesex, UK), according to [31]. Briefly, 48 h after inoculation, the HepG2 culture medium was replaced by fresh medium with CgAE and CgEE (9:1) at final concentrations ranging from 50 to 1000 µg/mL. After 48 h, the MTT assay was performed. The data were expressed as a percentage relative to the solvent control. Four replicates of each sample were performed, *n* = 2–3. The % cell viability was calculated according to the following formula:

Cellviability%=Abs1Abs0×100

where Abs1 is the absorbance of extract-treated cells and Abs0 is the absorbance of solvent control-treated cells.

### 4.10. Data Analysis

Statistical analysis was performed with STATISTICA version 7.0 (StatSoft Inc., Tulsa, OK, USA). The quantification of secondary metabolites, antioxidant activity, and inhibition of enzymes was tested by the Student *t*-test. Regression analyses were carried out using EXCEL (Microsoft Corporation, USA). Cytotoxicity data was analyzed with a two-way ANOVA and graphs plotted using GraphPad Prism^®^ software (version 9.0.0.121, GraphPad Software, San Diego, CA, USA). Results are presented as mean ± standard deviation; *p <* 0.05 was considered significant.

## 5. Conclusions

This study is the first report on micromorphology features, in vitro biological studies (antioxidant and antihyperglycemic potential), and the pre-clinical safety of *C. glaber* aerial parts used as an herbal drug in Cabo Verde. The characteristic micromorphological features identified together with the phytochemical profile are important for the quality control of this medicinal plant as they allow its correct identification and can be included in official quality monographs, which allow its pharmaceutical use as an herbal medicinal product or as part of herbal preparations for medicinal purposes. Our results showed that *C. glaber* aerial parts are a source of natural products with antioxidant and antihyperglycemic potential and possibly with multiple therapeutic and economic values. Furthermore, the data gathered in this study can serve as an incentive for the development of conservation strategies for this species, as it is a source of valuable natural products.

Future perspectives will include anti-inflammatory activity studies, according to its traditional use, and the isolation of selected promising compounds, to better understand the undelaying mechanisms of action of this medicinal plant.

## Figures and Tables

**Table 1:** Quantitative anatomical features of the *C. glaber* stem and leaf.

Anatomical Parameters	Min–Max	Mean	±SD
Stem			
Cork (no. of cell layers)	3–7	4	1
Sclereid cell wall thickness (µm)	3.3–10.2	6.8	2.1
Xylem vessel area (µm^2^)	82.1–324.1	208	74
Central cylinder cell area (µm^2^)	770.9–4340.5	2304	1024
Leaf			
Cuticule thickness (µm)	13.7–25.6	19.2	3.3
Pallissade parenchyma thickness (µm)	154–323	254.7	34.2
Epidermal cell length (µm)	22.1–55.4	36.4	7.9
Epidermal cell width (µm)	13.6–38.5	24.8	5.8
Stomata length (µm)	31.3–46.6	38.4	3.8
Stomata width (µm)	25.9–38.6	32.4	3.2
Stomata index	-	4.3	1.2
Idioblast area (µm^2^)	73.1–96.8	88.7	6.5
Thrichome stalk length (µm)	49.4–120.7	77.1	16.6

Max: maximum; Min: minimum; SD: standard deviation.

**Table 2:** HPLC-UV-ESI/MS-MS identification of *C. glaber* aerial parts main marker compounds.

Peak	*t*_r_ (min)	UV*λ*_max_ (nm)	[M-H]^−^	MS-MS Fragment Ions *m*/*z* (Relative Abundance)	Proposed Compound	Class
a	3.52	261	133	133 (88); 115 (100); 71(40); 73 (18)	Malic acid	Organic acid
b	4.89	269	191	191(45); 111(100); 87(38); 85 (27)	Citric acid	Organic acid
c	6.95	234	373	123 (100); 193(75); 149 (48)	Gardoside	Iridoid
d	9.05	238	373	123 (100); 149 (80)	Geniposidic acid *	Iridoid
e	11.95	235	375	169 (100); 89 (65); 151 (59); 213(50)	8-Epiloganic acid	Iridoid
f	13.82	244; 330	487	179 (100); 135(13); 161 (12); 341 (5)	Cistanoside derivative	Phenylethanoid glycoside
g	14.82	236	451	243(100); 101(50)	Iridoid derivative	Iridoid
h	20.32	283; 343	667	351 (100); 113 (12)	Unknown	Flavonoid
i	21.05	238; 304	453	89 (100); 159 (90)	Unknown	Unknown
j	21.65	246; 283sh; 324	639	639 (100); 621(33); 161(26); 179 (23); 451 (22)	*β*-Hydroxyverbascoside isomer	Phenylethanoid glycoside
k	24.59	283; 343	667	351 (100); 315 (28); 113 (25)	Unknown	Flavonoid
l	28.35	244; 284sh; 332	637	637 (100); 251 (70); 62(63); 623 (25)	Unknown	Phenylethanoid glycoside
m	28.99	331	755	755 (100); 161 (22); 593 (12)	Lavandulifolioside	Phenylethanoid glycoside
n	29.85	330	623	623 (100); 161 (28); 461 (12); 135 (3)	Verbascoside	Phenylethanoid glycoside
o	31.12	331	623	623 (100); 161 (28); 461 (12); 135 (3)	*cis*-Verbascoside	Phenylethanoid glycoside
p	34.72	328	623	623 (100); 161 (28); 461 (12); 135 (3)	Isoverbascoside	Phenylethanoid glycoside

* Identified with reference standard; [M-H]**^−^**—negative mass electrospray ionization mode; MS/MS—mass spectrometry; *m*/*z*—mass-/charge ratio; *t*_r_—retention time; UV *λ*_max_—wavelength of maximum absorbance.

**Table 3:** DER, total phenols, flavonoids, and iridoid contents of *C. glaber* aerial part extracts.

Plant Extracts	DER	TPC (mg GAE/g)	TFC (mg CE/g)	TIC (mg AuE/g)
CgAE	4.39:1	131.3 ^b^ ± 3.9	71.0 ^b^ ± 0.6	4.9 ^a^ ± 0.6
CgEE	2.89:1	148.1 ^a^ ± 9.5	75.8 ^a^ ± 0.2	6.1 ^a^ ± 1.3

CgAE: *Campylanthus glaber* aqueous extract; CgEE: *Campylanthus glaber* ethanolic extract; DER: drug–extract ratio; TPC: total polyphenolic content; TFC: total flavonoid content; TIC: total iridoid content; GAE: gallic acid equivalents; CE: catechin equivalents; AuE: aucubin equivalents. In each column, different letters denote significant differences (*p* < 0.05).

**Table 4:** CUPRAC, FRAP, and DPPH radical scavenging capacity of *C. glaber* aerial part extracts.

Samples	CUPRAC (µg AA/g)	FRAP (µg AA/g)	DPPH^•^ IC_50_ (µg/mL)
CgAE	197.9 ^b^ ± 2.6	109.8 ^a^ ± 3.2	130.9 ^a^ ± 1.4
CgEE	203.8 ^a^ ± 1.8	104.0 ^a^ ± 3.9	134.3 ^a^ ± 3.1
Ascorbic acid	-	-	17.3 ± 0.3

CgAE: *Campylanthus glaber* aqueous extract; CgEE: *Campylanthus glaber* ethanolic extract; CUPRAC: cupric-reducing antioxidant capacity; FRAP: ferric-reducing antioxidant power; DPPH**^•^**: 2,2-diphenyl-1-picrylhydrazyl radical; AA: ascorbic acid; IC_50_: half maximal inhibitory concentration. In each column, different letters denote significant differences (*p* < 0.05).

**Table 5:** *C. glaber* aerial part extract inhibitory enzymatic activity against *α*-amylase and *α*-glucosidase enzymes.

Samples	*α*-Amylase IC_50_ (mg/mL)	*α*-Glucosidase IC_50_ (µg/mL)
CgAE	7.21 ^c^ ± 0.23	nd
CgEE	5.77 ^b^ ± 0.10	827.9 ^b^ ± 11.2
Acarbose	0.011 ^a^ ± 0.001	350.3 ^a^ ± 15.4

CgAE: *Campylanthus glaber* aqueous extract; CgEE: *Campylanthus glaber* ethanolic extract; IC_50_: The half maximal inhibitory concentration. In each column different letters denote significant differences (*p* < 0.05); nd: not defined.

**Table 6:** Genotoxic potential of *C. glaber* aerial part extracted by the Ames test without metabolic activation.

CgAE (µg/Plate)	Revertant Colonies per Plate (Mean ± SD) without Metabolic Activation				
	TA98	TA100	TA102	TA1535	TA1537
0	26 ± 4	148 ± 6	361 ± 9	20 ± 4	28 ±5
250	26 ± 4	167 ± 20	358 ± 24	14 ± 1	31 ± 9
625	25 ± 6	166 ± 22	388 ± 17	16 ± 5	26 ± 6
1250	26 ± 2	154 ± 15	365 ± 20	22 ± 5	27 ± 6
2500	25 ± 2	162 ± 13	377 ± 19	16 ± 2	21 ± 1
3750	28 ± 2	170 ± 61	384 ± 21	20 ± 6	30 ± 2
5000	26 ± 2	178 ± 5	406 ± 35	14 ± 3	27 ± 8
CgEE (µg/plate)	Revertant colonies per plate (mean ± SD) without metabolic activation				
	TA98	TA100	TA102	TA1535	TA1537
0	26 ± 5	196 ± 5	344 ± 5	13 ± 3	25 ± 3
250	25 ± 5	179 ± 22	364 ± 5	14 ± 3	25 ± 3
625	25 ± 7	192 ± 22	361 ± 7	14 ± 1	28 ± 3
1250	26 ± 6	199 ± 6	347 ± 6	12 ± 1	26 ± 3
2500	29 ± 8	204 ± 8	391 ± 8	14 ± 3	21 ± 5
3750	28 ± 5	213 ± 22	388 ± 5	20 ± 7	25 ± 8
5000	30 ± 3	221 ± 6	388 ± 3	11 ± 1	24 ± 4
PC	(1)	(2)	(3)	(2)	(4)
	488 ± 30 *	1048 ± 43 *	881 ± 26 *	827 ± 13 *	1354 ± 5 *

CgAE: *Campylanthus glaber* aqueous extract; CgEE: *Campylanthus glaber* ethanolic extract; PC (positive control); SD (standard deviation); (1)—2-nitrofluorene; (2)—sodium azide; (3)—*tert*-butyl hydroperoxide; (4)—9-aminoacridine. * These values are similar to those obtained by Malmir et al. (2023) [31] and Malu et al. (2022) [32], since this study was performed simultaneously.

**Table 7:** Genotoxic potential of CgEE tested with *Salmonella* Typhimurium strains TA98, TA100, TA102, TA1535, and 1537 with metabolic activation.

CgEE (µg/Plate)	Revertant Colonies per Plate (Mean ± SD) with Metabolic Activation				
	TA98	TA100	TA102	TA1535	TA1537
0	47 ± 4	157 ± 6	172 ± 2	11 ± 2	12 ± 1
625	61 ± 7	146 ± 7	180 ± 35	12 ± 2	16 ± 3
1250	65 ± 4	167 ± 18	200 ± 11	16 ± 1	14 ± 3
2500	50 ± 6	175 ± 16	205 ± 3	16 ± 1	15 ± 2
5000	37 ± 3	162 ± 3	180 ± 35	12 ± 1	15 ± 4
PC	(1)	(2)	(1)	(1)	(1)
	832 ± 35 *	947 ± 148 *	732 ± 12 *	266 ± 1 *	306 ± 50 *

CgAE: *Campylanthus glaber* aqueous extract; CgEE: *Campylanthus glaber* ethanolic extract; PC (positive control); SD (standard deviation); (1)—2-aminoanthracene; (2)—benzo(a)pyrene. * These values are similar to those obtained by Malmir et al. (2023) [31] and Malu et al. (2022) [32], since this study was performed simultaneously.

## Data Availability

Data is contained within the article.
